# Increasing the utilisation of hydroxychloroquine blood level testing in lupus: a quality improvement project

**DOI:** 10.1136/lupus-2026-002113

**Published:** 2026-06-29

**Authors:** Zeinab Saleh, Elizabeth Spranger, Sarah Smeets-Parmelee, Joanne Michelle Kahlenberg, Carmen Gherasim, Catherine Slomiany, Maya Aravind, Roni M Shtein, Shqipe Selman, Rebeca Packard, Tammy Ellies, Anita Devine, Wendy Marder, Emily C Somers

**Affiliations:** 1 Departments of Internal Medicine-Rheumatology and Dermatology, University of Michigan, Ann Arbor, Michigan, USA; 2 Internal Medicine, University of Michigan, Ann Arbor, Michigan, USA; 3 Health Information Technology and Services, University of Michigan, Ann Arbor, Michigan, USA; 4 Pathology, University of Michigan, Ann Arbor, Michigan, USA; 5 Dermatology, University of Michigan, Ann Arbor, Michigan, USA; 6 Ophthalmology, University of Michigan, Ann Arbor, Michigan, USA; 7 Pharmacy, University of Michigan, Ann Arbor, Michigan, USA; 8 University of Michigan, Ann Arbor, Michigan, USA; 9 Departments of Internal Medicine-Rheumatology and Obstetrics and Gynecology, University of Michigan, Ann Arbor, Michigan, USA; 10 Departments of Internal Medicine-Rheumatology, Environmental Health Sciences, and Obstetrics and Gynecology, University of Michigan, Ann Arbor, Michigan, USA

**Keywords:** Systemic Lupus Erythematosus, Antirheumatic Agents, Quality Indicators, Health Care

## Abstract

**Objective:**

Hydroxychloroquine (HCQ) is a cornerstone therapy for SLE, reducing flares, organ damage and mortality. However, weight-based dosing yields highly variable drug exposure, which can result in suboptimal treatment. Measuring HCQ blood levels provides an objective tool to assess adherence and guide individualised dosing. Although available at our academic medical centre, this testing remains infrequently ordered in routine practice. We aimed to increase HCQ testing in patients with SLE through a structured quality improvement initiative and to characterise the clinical distribution of HCQ level results among tested patients.

**Methods:**

We conducted a single-centre quality improvement project from August 2024 to November 2025 using a Plan-Do-Study-Act framework. Interventions included multidisciplinary education, development of institutional guidelines and implementation of an order set incorporating HCQ testing at prescribing. The primary outcome was the proportion of eligible patients with SLE with HCQ testing. As a secondary analysis, HCQ level results were categorised into four clinically meaningful ranges (severe non-adherence, subtherapeutic, therapeutic and supratherapeutic) based on published literature.

**Results:**

Among nearly 1000 adult patients with SLE, HCQ testing increased from 4% to 15% (absolute increase of 11 percentage points; more than threefold relative). Provider adoption increased from 24% (8/33) to 73% (24/33) of rheumatologists. Among 170 tested patients, 79.4% had HCQ levels below the therapeutic range, including 14.7% with severe non-adherence (<200 ng/mL), approximately twice that in published cohorts.

**Conclusions:**

This real-world quality improvement initiative demonstrates both the feasibility and clinical yield of integrating HCQ monitoring into routine SLE care. Beyond increasing test utilisation, monitoring revealed a substantial burden of HCQ exposure below the therapeutic range that would have remained invisible without systematic testing. The model is readily adaptable to other centres, particularly those serving populations facing access barriers.

**WHAT IS ALREADY KNOWN ON THIS TOPIC**

- Hydroxychloroquine (HCQ) is standard-of-care therapy for SLE, and growing evidence supports blood level monitoring to objectively assess adherence and guide individualised dosing.
- Current evidence suggests that HCQ blood levels between 750 and 1150 ng/mL are associated with improved outcomes, while levels outside this range may reflect non-adherence, increased toxicity risk, or interindividual pharmacokinetic variability.
- Despite these data, HCQ blood level testing remains underutilised, in part because professional rheumatology societies have not yet incorporated it into formal guidelines.

**WHAT THIS STUDY ADDS**

- This is a pioneering quality improvement project that addresses the underutilisation of HCQ blood level testing in clinical practice.
- This study demonstrates that a structured initiative incorporating electronic health record workflow optimisation, institutional guideline development, and targeted education increased testing from 4% to 15% among nearly 1000 patients with SLE.
- Among tested patients, nearly 80% had HCQ blood levels below the therapeutic range, and 14.7% met criteria for severe non-adherence, approximately twice the rate reported in published cohorts, illustrating a substantial burden of suboptimal HCQ exposure in routine clinical practice that may otherwise go undetected.

**HOW THIS STUDY MIGHT AFFECT RESEARCH, PRACTICE OR POLICY**

- This initiative provides a scalable model for integrating HCQ blood level monitoring into routine SLE care that can be adapted by other institutions.
- By addressing the absence of consensus guidance, our study demonstrates that institution-level, evidence-based guidelines can operationalise HCQ blood level monitoring in clinical practice, with the potential to inform broader adoption and support the development of formal society guidelines.
- Our findings underscore that real-world rates of HCQ exposure below the therapeutic range may exceed those reported in published cohorts, supporting routine HCQ monitoring as a tool for individualised lupus care.

## Introduction

 SLE is a female-predominant autoimmune disease, with age-specific incidence rising during the reproductive years.^1^,^3^ Thus, management of SLE, including the need to balance risks and benefits of treatment options, often extends across much of a person’s lifespan.

Hydroxychloroquine (HCQ) is considered standard of care for patients with SLE, regardless of organ involvement or disease severity.^4^ HCQ is associated with reduced disease activity, morbidity and mortality in SLE, and due to its broad clinical benefits, its continuation with appropriate monitoring is advised, even during periods of clinical remission.^4^,^6^ Furthermore, HCQ is associated with benefits in terms of maternal and foetal outcomes^7^ and recommended in SLE during preconception, pregnancy and breastfeeding stages.^8^

Despite consensus regarding the benefits of HCQ in SLE, optimal dosing remains a source of contention due to concerns about irreversible retinal toxicity. To mitigate this risk, the American Academy of Ophthalmology (AAO) updated its guidelines in 2016, lowering the maximum daily HCQ dose from 6.5 mg/kg of ideal body weight to 5.0 mg/kg of real body weight,^9^ a recommendation that remains unchanged in the 2025 AAO guidelines.^10^ However, multiple studies have demonstrated that HCQ dosing at ≤5 mg/kg/day is associated with an increased risk of lupus flares,^11 12^ as well as higher rates of SLE-related hospitalisation.^13^ Collectively, these findings suggest that current guideline-recommended dosing may compromise disease control and underscore the need to carefully balance retinopathy risk against HCQ efficacy.

Beyond dosing, adherence presents a second major challenge. A substantial proportion (36%) of patients with SLE in the USA are persistently nonadherent to HCQ, and 47% are intermittently adherent.^14^ In southeastern Michigan, where our centre is based, 22% of patients with SLE report deviating from taking medication as prescribed to save money,^15^ and such cost-related nonadherence is associated with higher levels of SLE-related disease activity and irreversible organ damage.^16^

To help clinicians navigate the narrow margin between efficacy and toxicity, as well as objectively assess non-adherence or partial adherence, lupus researchers worldwide have proposed therapeutic HCQ blood level monitoring as a strategy to guide individualised dosing and guidance.^17^ Accumulating evidence has defined four clinically meaningful HCQ blood level ranges in SLE, although these thresholds remain preliminary and continued refinement is anticipated as additional data accrue: severe non-adherence (whole blood HCQ levels <200 ng/mL), reflecting frequent interruptions or discontinuation of therapy^18^,^23^; partial non-adherence/subtherapeutic (whole blood levels 200–749 ng/mL), reflecting intermittent missed doses and associated with higher risk of flares^24^; therapeutic (whole blood levels 750–1149 ng/mL), associated with reduced disease activity^17 23 25^ and supratherapeutic (whole blood levels ≥1150 ng/mL), associated with a 2.1-fold higher odds of toxicity without additional therapeutic benefit.^17 26^

Despite growing evidence demonstrating that HCQ monitoring improves patient outcomes and its routine implementation at some centres (particularly in France, some other European countries, and the USA), this test remains overall underutilised.^4^ One contributing factor is that HCQ testing has not yet been formally adopted by professional rheumatology societies, such as the American College of Rheumatology (ACR) or European Alliance of Associations of Rheumatology (EULAR), due to the need for further standardisation and broader availability.^4^ Accordingly, the primary aim of this quality improvement (QI) initiative was to assess baseline utilisation of HCQ blood level testing at the University of Michigan Health (U-M Health) and to implement system-level interventions to increase its use among patients with SLE.

## Methods

### Study design and setting

This QI project was conducted at U-M Health, a US-based academic medical centre, from 1 August 2024 to 1 November 2025. The project was implemented in three sequential phases. Phase 1 (1 August–30 September 2024) involved a current state assessment to characterise baseline practice patterns related to HCQ blood testing. Phase 2 (1 October 2024–30 April 2025) focused on performing a structured root-cause analysis and developing targeted interventions to address identified barriers. Phase 3 (1 May 2025–1 November 2025) consisted of the implementation of these interventions and the evaluation of their impact.

The multidisciplinary QI team included healthcare professionals with expertise in rheumatology (including lupus subspecialisation) (n=3), epidemiology (n=1), dermatology (n=1) and ophthalmology (n=1), as well as representatives from nursing (n=1), pharmacy (n=1), pathology (n=1), continuous improvement (n=3), data analytics (n=1), Health Information Technology and Services (n=1) and a patient advisor (n=1). The rheumatologist lead and one continuous improvement specialist met weekly for 30–60 min, while the full multidisciplinary team convened monthly for 1 hour.

Because the project focused on system-level practice improvement rather than human subjects research, it did not require individual informed consent and was determined as ‘not regulated’ by the University of Michigan Institutional Review Board (HUM00255412).

### Study population

The population of focus for this QI project included adults aged 18 years or older with SLE, identified through electronic health record (EHR) queries of problem lists or encounter diagnoses (International Classification of Diseases, 10th revision codes: M32.0, M32.1, M32.8 and M32.9). The ‘eligible’ population included patients with SLE (as defined above) who (1) were managed by the adult rheumatology service at U-M Health, (2) had at least two rheumatology encounters within the 18 months preceding the measurement date and (3) had an active HCQ prescription (defined as a prescription without an end date) as of the time of the measurement date. By design, the eligibility criteria captured the outpatient SLE population; inpatient HCQ ordering practices were not assessed.

### Measures

The primary process measure was the proportion of adult patients with SLE who had an HCQ blood level test performed as part of routine clinical care. HCQ concentrations accumulate progressively after treatment initiation until steady state is achieved, typically after five drug half-lives (approximately 4–6 months of consistent daily dosing).^27^ Therefore, we used a 6-month rolling window to assess the proportion of eligible patients who had an HCQ level test ordered. For example, the measurement on 1 August 2024 represented the proportion of patients who had a test performed within the 6 months preceding that date (February–August 2024). The denominator included all eligible patients during the relevant time window, and the numerator included those for whom an HCQ test was ordered during the defined time window. Measurements were conducted at the patient level rather than the visit level; thus, patients with multiple encounters within the defined time frame were counted only once to prevent duplication.

The secondary process measure was the number of rheumatology providers (out of 33 total) who ordered at least one HCQ test for their patients with SLE during the QI project.

As a secondary descriptive analysis, HCQ level results among all patients tested at least once during the implementation phase were categorised into four clinically meaningful ranges based on the published lupus literature^24 28^: severe non-adherence (<200 ng/mL), subtherapeutic (200–749 ng/mL), therapeutic (750–1149 ng/mL) and supratherapeutic (≥1150 ng/mL). HCQ levels were drawn as part of routine clinical visits without standardised timing relative to the most recent dose (random levels). Although our institutional laboratory reports HCQ concentrations as serum levels, results were converted to whole blood equivalents (whole blood≈serum÷0.53)^29^ for consistency with the published lupus literature.

### QI framework and data analysis

We applied the Plan-Do-Study-Act (PDSA) framework as the QI methodology to identify gaps in HCQ test utilisation, implement targeted interventions, monitor changes over time and iteratively refine project strategies.^30^

The number and proportion of eligible patients with an HCQ level ordered were assessed through monthly automated data queries, which eliminated the need for manual chart abstraction and minimised the risk of transcription errors. Because the primary aim was QI and practice evaluation, no formal statistical analyses were performed.

### Current state assessment

#### Baseline data (prior to QI interventions)

At our institution, HCQ testing was added to the clinical laboratory formulary in 2019. In August 2024, only 4% of adult patients with SLE had an HCQ level measured within the preceding 6 months.

#### Surveys

To better understand the current state of practice as of August 2024, we administered distinct, role-specific anonymous electronic surveys to rheumatology providers and nurses at our institution. The provider survey focused on perceived barriers to ordering HCQ testing, whereas the nurse survey assessed knowledge of HCQ testing and the frequency of patient enquiries related to this test.

Key takeaways from the current state surveys:

- Rheumatology providers:
  - Among surveyed providers, 70% were unaware of the availability of a laboratory test to measure HCQ levels at our institution.
  - 95% reported never ordering HCQ testing for their patients with SLE.
  - 50% expressed uncertainty regarding therapeutic and toxic HCQ blood level thresholds.
- Rheumatology nurses:
  - 80% of respondents reported uncertainty regarding HCQ blood level thresholds.
  - Nurses indicated that patients receiving HCQ rarely or never enquire about HCQ testing results during clinical communications.

### Root-cause analysis

**Figure 1:**
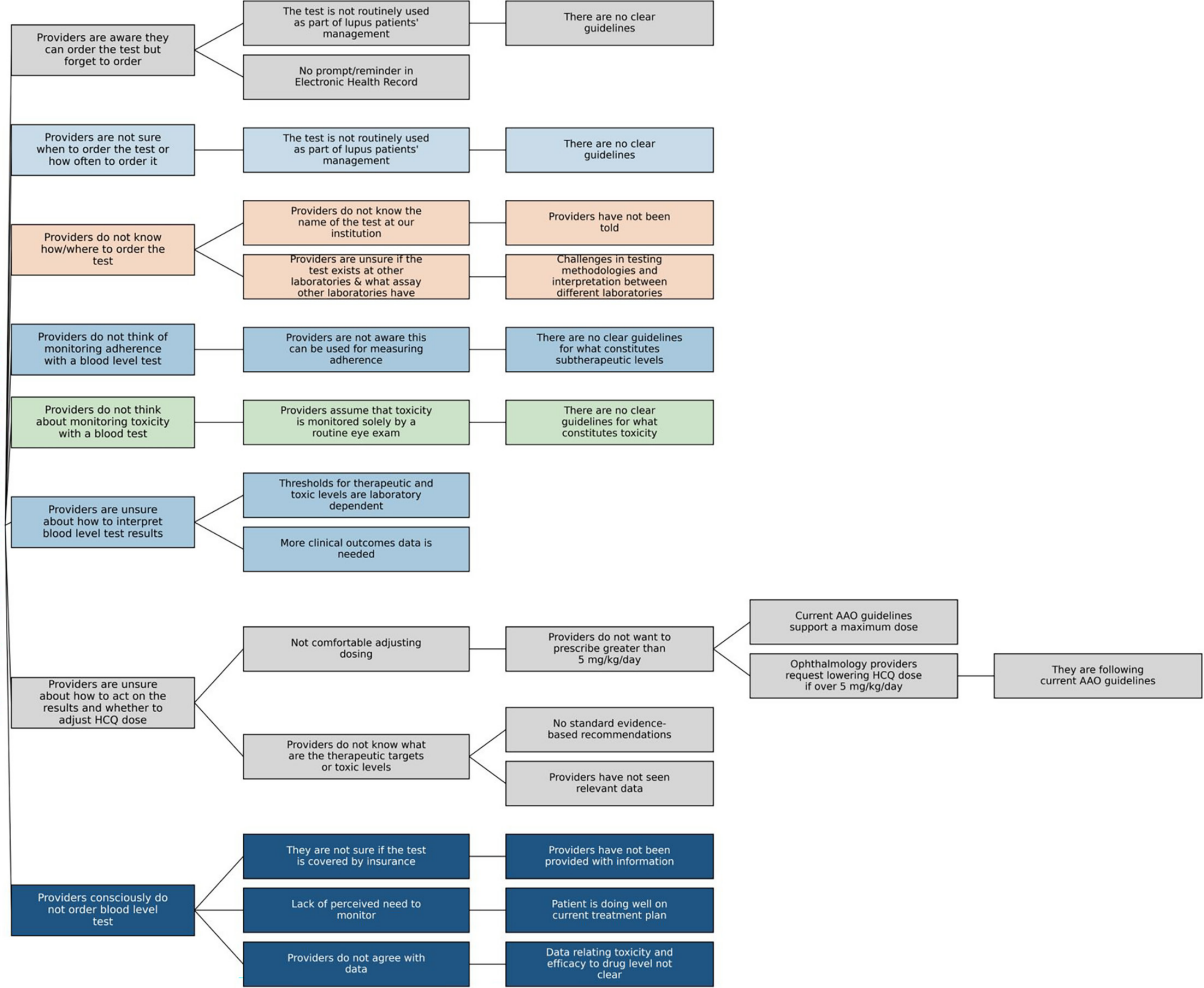
5 Whys root-cause analysis. AAO, American Academy of Ophthalmology; HCQ, hydroxychloroquine.

We performed a root-cause analysis using the ‘5 Whys’ method, a structured problem-solving technique designed to move beyond surface-level observations and identify underlying key drivers contributing to the underutilisation of HCQ testing (figure 1).^31^ The main causes for test underutilisation identified were the following.

1. Absence of professional society guidelines addressing HCQ monitoring.
2. Provider-level barriers, including failure or forgetfulness to order the test during routine clinical encounters.
3. Knowledge gaps and clinical uncertainty among providers regarding optimal time for test ordering, result interpretation and subsequent management decisions.

### QI interventions

Guided by the root-cause analysis and using an impact-effort matrix,^32^ we developed targeted interventions to address each identified key driver of underutilisation, prioritising low-effort, high-impact opportunities (online supplemental figure 1).

**Figure 2:**
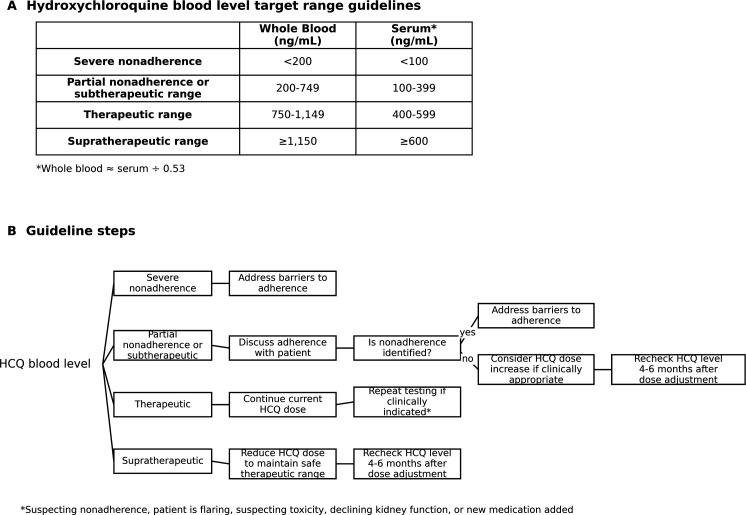
Institutional guidelines for monitoring HCQ blood levels. (**A**) Whole blood and serum target range definitions across clinical categories. (**B**) Decision algorithm for clinical interpretation and management based on blood level results. HCQ, hydroxychloroquine.

1. Development of institutional guidance: In the absence of formal evidence-based guidelines on HCQ testing from professional rheumatology associations, our team conducted a comprehensive literature review, held six multidisciplinary teleconferences and presented the available evidence at our rheumatology grand rounds. Through this iterative consensus-building process, we developed institutional recommendations for HCQ monitoring (figure 2). These guidelines were disseminated via PolicyStat, U-M Health’s centralised online repository for institutional policies and clinical guidelines and provided practical recommendations regarding appropriate indications for HCQ testing, result interpretation and subsequent clinical management.
2. EHR-based workflow optimisation: An EHR-based order set was implemented to incorporate HCQ testing following a new HCQ prescription. Additionally, during HCQ refill encounters, nursing staff prompted prescribing clinicians using a standardised text block (DotPhrase) to determine whether HCQ testing should be ordered. Providers could opt out for non-SLE indications or testing already done within the preceding 6 months.
3. Provider education: Education on the clinical importance of HCQ monitoring was delivered during monthly faculty meetings. Providers were oriented to the EHR order set and DotPhrase workflow and received periodic reminder emails reinforcing test availability, appropriate ordering and result interpretation. As part of these educational efforts, we clarified that HCQ testing is covered by most insurance providers in the USA and is supported by the established Current Procedural Terminology (CPT) code 80220, which supports its integration into routine clinical practice.
4. Nursing education: Rheumatology nurses received tailored educational materials and a 1-hour interactive session covering the clinical rationale for HCQ testing and provider recommendations. Nurses also received periodic reminder emails on result interpretation.
5. Patient education: With our patient advisor, we developed educational materials explaining the rationale for HCQ testing for use during clinical encounters to support shared decision-making.

### Patient and public involvement

A patient advisor participated as a member of the multidisciplinary team during the planning and implementation phases, providing patient-centred perspectives that informed intervention design, particularly the development of patient-facing educational materials to support understanding of HCQ testing. Patients were not involved in defining research questions, selecting outcome measures or recruitment, as this was a system-level QI initiative using existing clinical data.

## Results

**Figure 3:**
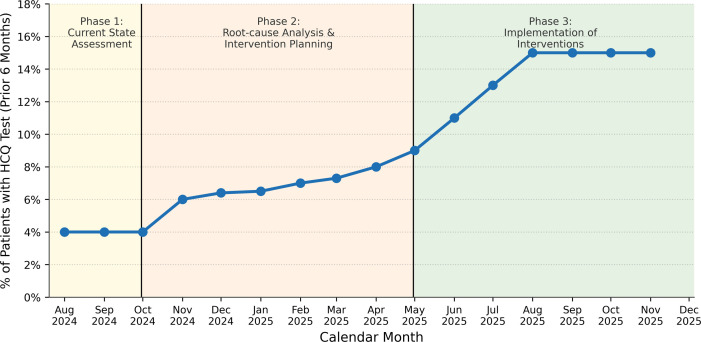
Percentage of lupus patients with hydroxychloroquine blood level testing over time, based on 6-month rolling windows. Each data point represents the percentage of patients tested within the preceding 6 months. Coloured panels denote the three project phases: Phase 1 (current state assessment), Phase 2 (root-cause analysis and intervention planning) and Phase 3 (implementation of interventions). HCQ, hydroxychloroquine.

Over the project period, HCQ testing increased from 4% (46/1065) to 15% (179/1157), and provider adoption expanded from 8 to 24 of 33 rheumatologists (figure 3 and table 1). Notably, this rise was not confined to the postintervention phase: HCQ testing increased from 4% to 9% during Phases 1 and 2, prior to deployment of the formal intervention bundle in Phase 3, with provider adoption expanding from 8 to 21 clinicians. Following intervention deployment, ordering increased further from 9% to 15%, representing an additional 6 percentage point absolute increase, with provider adoption expanding from 21 to 24 clinicians (figure 3 and table 1).

**Table 1:** Summary of process measures

Date	8/2024	5/2025	11/2025
Number (%) of lupus patients with HCQ blood level ordered in the preceding 6 months	46/1065 (4%)	103/1110 (9%)	179/1157 (15%)
Number (%) of rheumatology providers ordering HCQ blood level by this date	8/33 (24%)	21/33 (64%)	24/33 (73%)

HCQ, hydroxychloroquine.

Among the 179 patients with at least one HCQ level ordered during the project period, 170 had result values available (table 2). The majority of tested patients (135, 79.4%) had levels below the therapeutic range, with 25 (14.7%) classified as severe non-adherence and 110 (64.7%) subtherapeutic. Only 25 patients (14.7%) were within the therapeutic range, while 10 (5.9%) had supratherapeutic levels.

**Table 2:** Distribution of HCQ level results among tested patients, by clinical category

Clinical category	Proposed HCQ blood level range*	N (%)
Severe nonadherence	<200 ng/mL	25 (14.7)†
Subtherapeutic	200–749 ng/mL	110 (64.7)
Therapeutic	750–1149 ng/mL	25 (14.7)
Supratherapeutic	≥1150 ng/mL	10 (5.9)
Total	All ranges	170 (100)

* Proposed thresholds based on the published literature; these ranges remain preliminary and subject to ongoing validation.

† Includes one non-numeric result reported as ‘<10 ng/mL’, classified as severe non-adherence based on its position below the assay’s lower limit of detection.

HCQ, hydroxychloroquine.

## Discussion

### Process measure improvements

This QI initiative resulted in an absolute increase of 11 percentage points (from 4% to 15%) in HCQ testing among patients with SLE, representing more than a threefold relative increase. Notably, provider adoption showed a more pronounced shift, with the proportion of rheumatologists ordering at least one HCQ level increasing from 24% (8/33) to 73% (24/33), an absolute increase of 49 percentage points, suggesting broad practice adoption rather than behaviour change driven by a few clinicians.

As described in the Results section, approximately half of the testing increase occurred during Phases 1 and 2 prior to formal intervention deployment, driven by provider engagement in surveys, root-cause analysis discussions, and educational presentations during the planning phase. This preintervention improvement makes it difficult to attribute the overall increase solely to the formal intervention bundle, as awareness-raising activities and structured interventions are inherently intertwined in QI initiatives of this design. The rate of increase in ordering nearly doubled following formal intervention deployment (approximately 1.0 vs 0.6 percentage points per month), suggesting that the structured interventions amplified the practice change beyond what was achieved through awareness-raising alone. These subsequent gains underscore the added value of system-level strategies, including EHR-based workflow integration and institutional guidelines, in embedding practice change into workflows rather than relying on individual provider awareness alone, which is difficult to sustain over time.

Despite these gains, 85% of eligible patients had not yet been tested by the end of the project period, and the primary process measure plateaued at approximately 15% by August 2025 (figure 3). Multiple factors likely contributed to this plateau, including seasonal workflow disruptions, onboarding of new trainees and competing clinical priorities at the start of the academic year, as well as the broader evidentiary uncertainties addressed in the ‘Persistent challenges’ section below. Nevertheless, the breadth of provider engagement suggests that the infrastructure and awareness necessary for sustained adoption are now in place.

### Distribution of HCQ blood levels and clinical implications

Beyond process-level gains, our findings, drawn from routine clinical care rather than a structured research cohort, provide a window into the real-world distribution of HCQ levels and reveal clinically meaningful patterns. Nearly 80% of tested patients had levels below the therapeutic range, including 14.7% with severe non-adherence (<200 ng/mL) and 64.7% with subtherapeutic levels (200–749 ng/mL). Severe non-adherence at our centre is notably higher than the 7%–10% reported in published lupus cohorts,^28^ possibly reflecting the cost-related medication non-adherence previously documented in southeastern Michigan, where 22% of patients with SLE have reported deviating from prescribed regimens to save money.^15^ In the Systemic Lupus International Collaborating Clinics inception cohort, severe non-adherence was independently associated with higher risks of flare, damage accrual and mortality, underscoring the clinical importance of identifying these patients.^33^ Taken together, these observations position HCQ monitoring as both a tool for individualised care and an equity-informed strategy, capable of surfacing adherence barriers that disproportionately affect populations facing financial or access constraints; these barriers would otherwise remain invisible in routine clinical practice.

The proportion of patients with HCQ levels at or above the therapeutic threshold in our cohort (20.6%) was lower than the approximately 55% reported in a recent prospective cohort using contemporary weight-based dosing (mean 4.25 mg/kg/day, with 79% receiving ≤5 mg/kg/day).^34^ Because that cohort employed low-dose regimens comparable to current practice yet still achieved substantially higher therapeutic attainment, this difference is unlikely to be explained by reduced dosing under current ophthalmology guidelines. It more likely reflects the elevated non-adherence burden documented in our population, together with differences between an enrolled, repeatedly monitored longitudinal cohort and an unselected real-world clinical population identified through an EHR query. Of note, Durcan *et al* previously demonstrated that the proportion of patients with therapeutic HCQ levels increased substantially with repeated monitoring and counselling,^35^ suggesting that sustained HCQ monitoring at our institution may similarly improve the level distribution over time.

These findings reinforce the clinical relevance of our QI initiative in unmasking non-adherence that may otherwise go undetected and identifying a clinically actionable subgroup who may benefit from targeted adherence support, dose optimisation or further pharmacokinetic evaluation. Importantly, subtherapeutic levels cannot be attributed to adherence alone, as interindividual pharmacokinetic variability may also contribute, highlighting the value of integrating HCQ monitoring into individualised SLE care.

### Future directions: next PDSA cycles

Guided by the PDSA framework, the next phase of this initiative will focus on identifying barriers and facilitators to address the observed implementation plateau.

1. First, we plan targeted outreach to rheumatology providers who had not ordered HCQ testing (9 of 33) to better understand their perspectives and barriers and inform refinement of subsequent interventions. We anticipate that one-on-one educational outreach visits with these providers will help provide personalised, evidence-based support, consistent with prior evidence supporting academic detailing.^36^
2. Second, we will implement performance dashboards displaying real-time provider-specific HCQ monitoring rates alongside peer comparisons. Such dashboards have been shown to reduce clinical variation and influence ordering behaviour, with one study demonstrating a 17.3% reduction in undesired variation following implementation.^37^
3. Third, our planned reinforcement strategies include periodic reminders during faculty meetings, particularly during high-risk transition periods and targeted electronic communications to sustain engagement.
4. Fourth, we aim to broaden collaboration beyond rheumatology by engaging internal medicine and subspecialties involved in lupus care, including nephrology, neurology and pulmonary medicine, among others, as multidisciplinary approaches have been shown to enhance guideline implementation and uptake.^38^

Additional patient-directed strategies under consideration include automated text messaging and patient portal tools displaying individual HCQ levels and longitudinal trends to enhance engagement.

Beyond expanding test utilisation, future PDSA cycles will examine the downstream clinical impact of monitoring. This includes prescriber engagement with results, particularly the clinical actions taken following identification of severe nonadherence, subtherapeutic or supratherapeutic levels. It also includes correlation of HCQ levels with clinical outcomes such as flares and adherence patterns. Quantifying how monitoring translates into clinical action and outcomes will help characterise the clinical value of the QI initiative and identify opportunities to standardise evidence-based responses to actionable findings.

### Persistent challenges beyond local QI: implementation complexities in an evolving evidence base

A key challenge to our initiative was the absence of clear, consensus-based recommendations from professional rheumatology societies to guide clinicians in the routine use of HCQ monitoring, despite substantial evidence supporting its clinical benefits. Several practical implementation questions remain unresolved, each reflecting an underlying evidence base that is still maturing.

First, while extremely low HCQ levels are clinically informative and reflect non-adherence, thresholds defining the therapeutic and supratherapeutic ranges remain under validation, which has limited routine clinical implementation.^4^ More broadly, the supporting evidence base carries several caveats, including methodological limitations of existing pharmacokinetic studies, the predominantly observational nature of the available evidence and conceptual uncertainty about whether a single therapeutic window applies across patients and disease mechanisms.^28^ Second, HCQ levels are measured across laboratories using different biological matrices (whole blood vs serum) and analytical methodologies (high-performance liquid chromatography) or liquid chromatography with tandem mass spectrometry), complicating cross-institutional comparison and threshold standardisation.^24^ Third, preanalytical factors such as the timing of the last dose also affect interpretation. Random HCQ levels should be read with caution, given approximately 30% within-day variability between peak and trough.^24^ Although levels measured 6–8 hours after the last dose would ideally refine interpretation, this is difficult to achieve in routine clinical practice.^28^ Fourth, optimal patient selection remains debated; while any patient on HCQ may benefit from monitoring, the strongest evidence supports testing in selected scenarios such as disease flares, suspected non-adherence, toxicity risk factors, kidney impairment, pregnancy and obesity.^28^

These uncertainties have likely left many clinicians unfamiliar with implementation details (such as test ordering, monitoring frequency and result interpretation), tempered provider adoption at our institution and contributed to the observed plateau. This suggests that their caution reflects engagement with an evolving evidence base rather than knowledge gaps alone.

Recognising both the practical knowledge gaps and these evolving scientific questions, our multidisciplinary team developed U-M Health guidelines grounded in the most current evidence, with the understanding that recommendations will be iteratively updated as the field matures. These guidelines, designed to standardise practice at our centre, provide target HCQ blood level ranges for various clinical scenarios and a structured, stepwise approach for integrating HCQ levels into clinical workflows (figure 2). By disseminating our institutional experience, we aim to facilitate broader adoption of HCQ testing in routine SLE care. Combined with continued research, this broader adoption may support consensus-based recommendations and eventual incorporation of HCQ levels into professional society guidelines, creating a self-reinforcing cycle that accelerates uptake and improves clinical practice.

### Limitations

The initiative was conducted at a single U.S.-based academic tertiary care centre with established infrastructure, including an integrated EHR, a clinical laboratory offering HCQ testing since 2019, a centralised policy repository and dedicated continuous improvement resources. These features may limit direct generalisability to community rheumatology practices, smaller health systems or settings without access to HCQ testing or comparable EHR and QI infrastructure. Our southeastern Michigan patient population, with documented cost-related medication nonadherence, may differ from those served by other centres. Nevertheless, the structured, multi-component approach and reliance on widely available implementation strategies support adaptability to other institutional settings.

A second limitation relates to our choice of assessment window. As noted above, consensus on optimal HCQ monitoring frequency is lacking,^24^ and in routine practice, repeat testing is often deferred for patients with stable therapeutic levels, so reliance on a fixed 6-month assessment window may underestimate appropriate monitoring and artificially lower the proportion of patients classified as having undergone HCQ testing. Future work using longer windows or rolling look-back periods (eg, whether HCQ levels have ever been measured since initiation) may better capture clinically appropriate monitoring practices, particularly among stable patients. These challenges highlight a broader need for consensus guidelines on HCQ monitoring frequency.

A third limitation is that, as a system-level QI project focused on test utilisation, we did not capture individual-level clinical data. These include disease activity, flare status, the specific indication for each test order and prescriber actions taken in response to results (eg, adherence-focused conversations, dose adjustments or follow-up testing).

A fourth limitation is that HCQ levels in our cohort were drawn without standardised timing relative to the most recent dose. Because random levels can vary by approximately 30% between peak and trough, individual patients may be misclassified in either direction relative to their average HCQ exposure. However, severe non-adherence (<200 ng/mL) is unlikely to be explained by timing variability alone, and our central finding of a substantial unmet need for adherence support remains robust. Standardised timing of HCQ blood draws, for example, 6–8 hours post-dose, is a refinement worth exploring in future PDSA cycles.

## Conclusion

This real-world QI initiative demonstrates both the feasibility and clinical yield of integrating HCQ monitoring into routine SLE care. Through targeted interventions, testing among eligible patients increased from 4% to 15%, and provider adoption broadened from 24% to 73% of rheumatology clinicians. Beyond these process gains, testing surfaced a substantial clinical burden: nearly 80% of tested patients had HCQ levels below the therapeutic range, including 14.7% with severe nonadherence, roughly double that reported in published cohorts. This burden likely reflects the realities of routine clinical practice, including the cost-related medication non-adherence documented in southeastern Michigan, and would have remained largely invisible without systematic monitoring.

Our findings reinforce that HCQ monitoring is not merely a quality metric but a clinically meaningful intervention that identifies an actionable population for whom adherence support, dose optimisation or further pharmacokinetic evaluation may improve care. Because this model requires no incremental institutional resources, it is readily adaptable to other centres, particularly those serving populations facing cost-related medication nonadherence and other access barriers. Future PDSA cycles will sustain these gains, broaden monitoring across SLE care and translate identified levels below the therapeutic range into measurable clinical action and outcomes.

## Supplementary material

10.1136/lupus-2026-002113 online supplemental figure 1

## Data Availability

Data are available upon reasonable request.
