# Supplementary figures and images for: Increasing the utilisation of hydroxychloroquine blood level testing in lupus: a quality improvement project
Source: Lupus Sci Med. 2026 Jun 29;13(1):e002113. doi: 10.1136/lupus-2026-002113 (PMC13331187; doi:10.1136/lupus-2026-002113)

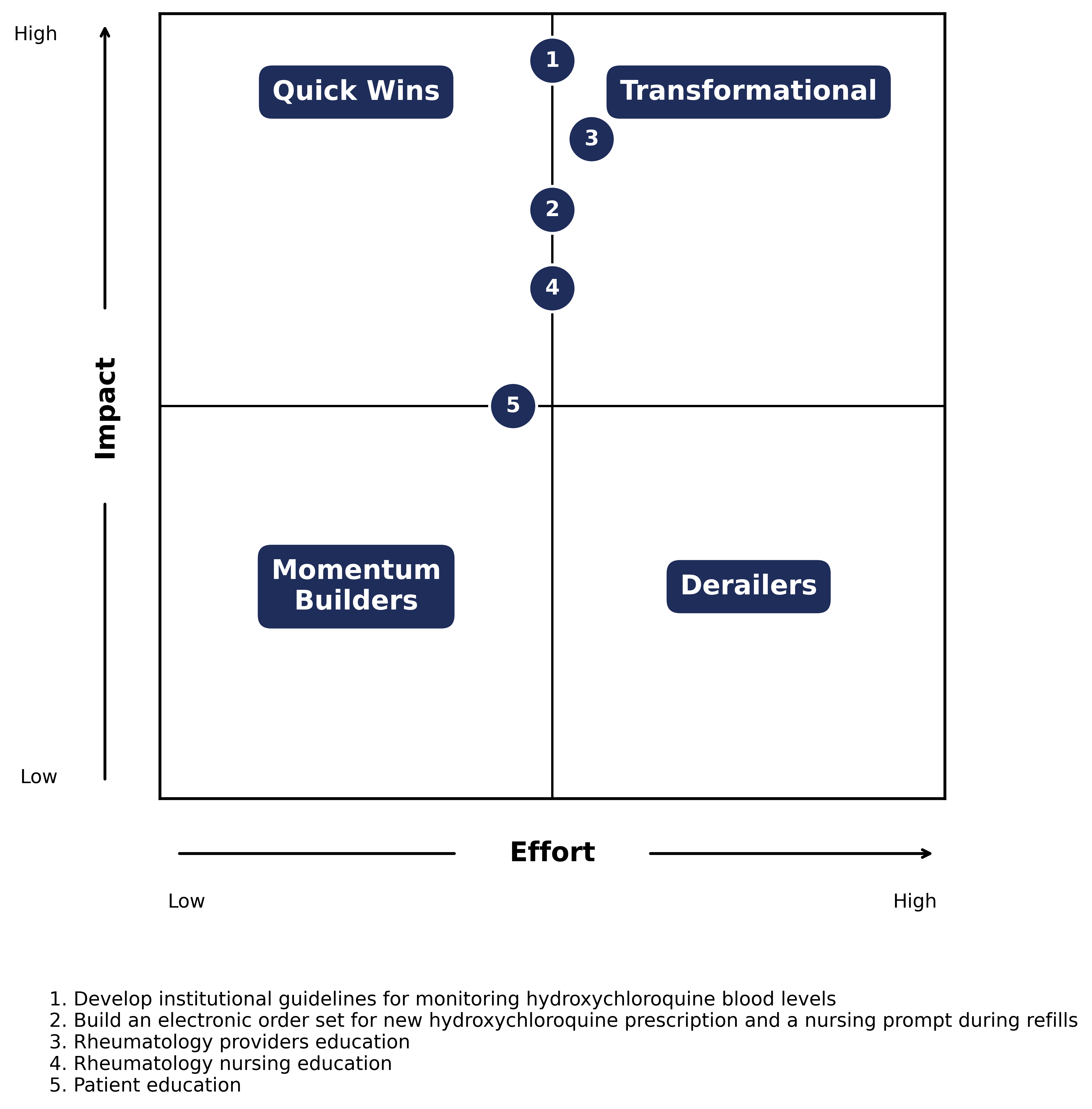

Supplement: online supplemental figure 1 [file lupus-13-1-s001.tif]
